# Identification of canine parvovirus with the Q370R point mutation in the VP2 gene from a giant panda (Ailuropoda melanoleuca)

**DOI:** 10.1186/1743-422X-10-163

**Published:** 2013-05-26

**Authors:** Ling Guo, Shao-lin Yang, Shi-jie Chen, Zhihe Zhang, Chengdong Wang, Rong Hou, Yupeng Ren, Xintian wen, Sanjie Cao, Wanzhu Guo, Zhongxiang Hao, Zifang Quan, Manli Zhang, Qi-gui Yan

**Affiliations:** 1 College of Veterinary Medicine, Sichuan Agricultural University, Ya’an, China; 2 Entry-exit Inspection and Quarantine Bureau, Chengdu, China; 3 Chengdu Research Base for Giant Panda Breeding, Chengdu, China; 4 Southwest University for Nationalities, Chengdu, Sichuan, China; 5 Key laboratory of Animal Disease and Human Health of Sichuan Province, Sichuan Agricultural University, Ya’an, China

**Keywords:** Canine parvovirus, VP2 gene, Point mutation, Phylogenetic analysis, Giant panda

## Abstract

**Background:**

In this study, we sequenced and phylogenetic analyses of the VP2 genes from twelve canine parvovirus (CPV) strains obtained from eleven domestic dogs and a giant panda (*Ailuropoda melanoleuca*) in China. A novel canine parvovirus (CPV) was detected from the giant panda in China.

**Results:**

Nucleotide and phylogenetic analysis of the capsid protein VP2 gene classified the CPV as a new CPV-2a type. Substitution of Gln for Arg at the conserved 370 residue in CPV presents an unusual variation in the new CPV-2a amino acid sequence of the giant panda and is further evidence for the continuing evolution of the virus.

**Conclusions:**

These findings extend the knowledge on CPV molecular epidemiology of particular relevance to wild carnivores.

## Background

Canine parvovirus type 2 (CPV-2) is an important pathogen in domestic dogs and several wild carnivore species. It was first identified in USA in 1978 and was found later to have spread worldwide in domestic and wild canine populations. CPV-2 is a small non-enveloped, singe-stranded DNA virus (5.2 Kb) which is a member of the genus *Parvovirus* of the family *Parvoviridae*. The CPV genome is composed of two major ORFs, one encoding the two non-structural proteins [NS1 and NS2] and the other encoding the two capsid proteins [VP1 and VP2] [1]. There is also a third protein, VP3 which is produced by proteolytic processing of VP2.

CPV-2 is antigenically and genetically related to feline panleukopenia virus (FPLV). However, FPV infects cats, mink and raccoons, but not dogs, whereas CPV-2 infects dogs and other *Canidae*, but not cats. A few amino acid differences between CPV and FPV determine the specificity of these viruses [2]. After the CPV-2 initial appearance (during 1978–1981), two new antigenic variants, named CPV-2a and CPV-2b, were characterized [3-5]. The antigenic types CPV-2a and CPV-2b differ from the original CPV-2 in at least five or six amino acids/residues of the VP2 capsid protein (genomic positions 3045, 3685, 3699, 4062 and 4449) [6,7]. Canine parvovirus type 2a/2b having mutation at 297 residue (Ser→Ala) is designated as new CPV-2a/2b [8,9], residue 297 is located in a minor antigenic site close to epitope B and substitutions at this position may be responsible for changes in antigenicity of CPV variants [10]. Another antigenic variant having an amino acid substitution 426-Asp→Glu was reported for the first time in Italy [11] and had been reported from other countries [1,12-17], and this variant is currently named as CPV-2c.

It has been reported that canine parvovirus (CPV) have been implicated in disease and mortality in giant pandas [18-21], which is an endangered species native to the China. The giant pandas with CPV infection showed diarrhea, vomiting and water-like feces [18]. Giant panda parvovirus VP2 gene described here identifies yet another variant of the virus. It demonstrates the continued adaptation of the virus to an everexpanding host range that includes endangered species of wildlife. Understanding emergent disease theats is important in enabling effective conservation measures for endangered species.

## Results

Out of 36 faecal samples of giant pandas and 97 canine rectal swabs screened by PCR assay using Hfor/Hrev primers, 1 giant panda and 62 dog samples yielded a specific amplicon of 611 bp, respectively.

The amplified PCR products of 11 randomly selected canine samples and one giant panda sample were subjected for sequencing using primer pair Hfor/Hrev. Primer pair Hfor/Hrev [11] encompasses informative amino acid residues which are of significance in characterizing the CPV types. All the CPV samples under study were found to be new CPV-2a (CPV-2a with nucleotide variation T→G at position 3675 or CPV-2a with amino acid variation 297-Ser→Ala). In comparison to prototype new-CPV-2a (AY742953), the samples under this study had amino acid residue variations at Tyr324Ile caused by mutation TAT →ATT at nt 3756–3758 of the VP2 gene. It was a unique mutation within the VP2 of Chinese and Korean strains of “new CPV-2a”. Critical positions of the CPV VP2 gene products of samples sequenced in this study are summarized in Table 1.

**Table 1:** CPV strains from China, origin from which they were isolated and their GenBank accession numbers

**Serial no**	**Strain**	**Host**	**Foster mode**	**Vaccinated**	**Origins**	**GenBank no.**
1	A10	Dog	Captive	Yes	Rectal swabs	JX624761
2	A11	Dog	Captive	Yes	Rectal swabs	JX624762
3	A12	Dog	Captive	Yes	Rectal swabs	JX624763
4	B01	Dog	Captive	Yes	Rectal swabs	JX624764
5	B02	Dog	Captive	Yes	Rectal swabs	JX624765
6	B03	Dog	Captive	Yes	Rectal swabs	JX624766
7	B04	Dog	Captive	Yes	Rectal swabs	JX624767
8	B05	Dog	Captive	Yes	Rectal swabs	JX624768
9	B06	Dog	Captive	Yes	Rectal swabs	JX624769
10	B07	Dog	Captive	Yes	Rectal swabs	JX624770
11	B11	Giant panda	Captive	Yes	Faecal	JX624771
12	B12	Dog	Captive	Yes	Rectal swabs	JX624772

In addition to the nucleotide variations at positions 3675 and 3756, three additional mutations were observed in the canine parvovirus sequences under study. One was at nucleotide position 3584 where a mutation (U→A) resulting in the codon change from UUC→UAC, with amino acid variation 267-Phe→Tyr. All the sequences under this study except B03, B06 and B11 showed this variation. The second one was at nucleotide position 4110, where variation A→G was observed and which changed the codon from ACG→GCG, with amino acid variation 442-Thr→Ala. This variation (A→G) at nucleotide position 4110 was observed in strains A10, A11, A12, B01, B02, B05, B07 and B12 in this study (the dog samples). The last mutation was at nucleotide position 3894 where a mutation (A→G) resulting in the codon change from CAA→CGA, with amino acid variation 370-Gln→Arg. This variation only was revealed in strain B11 (the giant panda sample).

**Figure 1:**
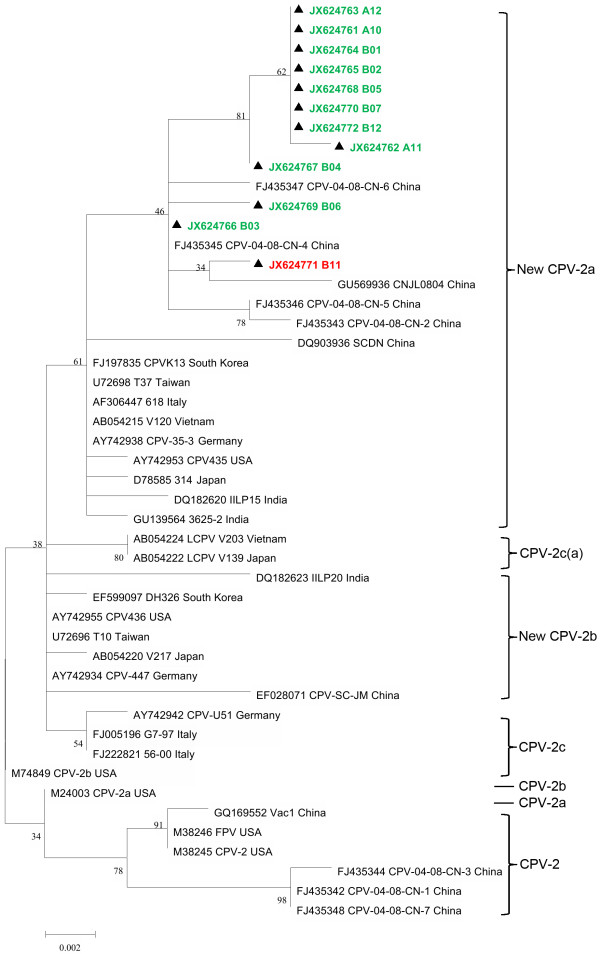
**Maximum likelihood tree (Mega-5.1 beta version) constructed using canine parvovirus sequences under study and the reference sequences.** M38246 (FPV, USA), M38245 (CPV-2, USA), M24003 (CPV-2a, USA), M74849 (CPV-2b, USA), AY742953 (New CPV-2a, USA), AY742955 (New CPV-2b, USA), DQ182620 (New CPV-2a, India), DQ182623 (New CPV-2b, India), D78585 (New CPV-2a, Japan), AB054220 (New CPV-2b, Japan), AY742938 (New CPV-2a, Germany), AY742934 (New CPV-2b, Germany), AY742942 (CPV-2c, Germany), AF306447 (New CPV-2a, Italy), AF306444 (CPV-2b, Italy), FJ222821 (CPV-2c, Italy), AB054222 (CPV-2c(a), Japan), AB054215 (New CPV-2a, Vietnam), AB054224 (CPV-2c(a), Vietnam), U72698 (New CPV-2a, Taiwan), U72696 (New CPV-2b, Taiwan), FJ197835 (New CPV-2a, South Korea), EF599097 (New CPV-2b, South Korea), DQ903936 (New CPV-2a, China), FJ435343 (New CPV-2a, China), FJ435342 (CPV-2, China), FJ435344 (CPV-2, China), FJ435345 (New CPV-2a, China), FJ435346 (New CPV-2a, China), FJ435347 (New CPV-2a, China), FJ435348 (CPV-2, China), EF599097 (New CPV-2b,South Korea), AB054215 (New CPV-2a, Japan), EF028071 (New CPV-2b, China), GQ169552 (CPV-2, China), Triangle (▲) indicates the 12 Chinese wild-type CPV strains analyzed in this study. Green and red were used for dog and giant panda strains, respectively.

To analyse the phylogenetic relationships of the China isolates with other CPV strains isolated in various parts of the world, we constructed a maximum likelihood phylogenetic tree. The panda field isolate B11 was found to be phylogenetically closely related to new CPV-2a strains of Jilin strain CNJL0804. B06 and B03 are the closest of Chinese dog sequences examined in this study. Rest of the other sequences had distinct lineage but shared molecular relationship with new CPV-2a reference strains (Figure 1).

## Discussion

New CPV-2a/2b appear to have replaced the prototype CPV-2a/2b strains and become the predominant types in many countries [22-26]. In China, the prevalent antigenic type in canine populations is type 2a, our results seem to confirm these data [27]. The dog and giant panda samples were all new CPV-2a, but it is impossible to conclude that new CPV-2a is predominant strain in the China dog or giant panda population because our data are limited to an exiguous number of samples and we do not have data regarding CPV-2 dog strains from the area where the giant panda samples were collected.

All the CPV clinical samples under study were found to be new CPV-2a (Figure 1). Similar substitution 297-Ser→Ala in CPV-2a strain were reported in VP2 gene of CPV from worldwide [2,22,28-30]. This study detected one site mutation among the CPV-2a isolates in China: Tyr→Ile at position 324, This mutation was also reported in 2009 [31]. Previous studies have shown that residue 324 is subject to strong positive selection in all parvoviruses of carnivores [32].

The natural adaptation of a virus to a new host is a very rare event, suggesting that there are high barriers that prevent viruses from gaining the ability to infect and spread naturally in hosts to which they are not adapted. In this study, the novel point mutation virus is most likely not a panda-adapted virus spreading among pandas, but more likely a spill-over from dogs. Because host adaptation involved complex interactions among both surface-exposed and buried capsid mutations that together altered cell infection and immune escape properties of the viruses [33]. In this study, the virus VP2 gene coding change at nt 3894 (VP2 residue 370, Gln→Arg) was interesting because it has not been detected previously in any other strains. We speculated that the mutation has two aspect functions. On the one hand, residue 370 was close to residues 375 and 377, it has indicated that residues 375 and 377 are associated with the ability of CPV to hemagglutinate or alter pH dependence of hemagglutination [34], On the other hand, residue 370 was adjacent to residue 379 and 384, while the 379 and 384 residue affects canine transferrin receptor (TfR) binding to determined the canine host range [35]. So, we made the inferences that R370 might be involved in a required conformational change, or it might mediate an effect on receptor binding through the neighboring residues, and is likely to have had an effect on the parvovirus host range. This observation suggests that the glutamine to arginine mutation may also affect host DNA±protein interaction. As is the case for wild animal, this mutation is not selected for the population, but may have arisen independently from various backgrounds.

As the phylogenetic tree shows, most of the viruses isolated in China formed a large cluster, while some strains clustered together with viruses from regions outside China. Most of the CPVs isolated in China formed a specific cluster and certain mutations detected in Chinese CPVs probably arose during the process of local adaptation, as indicated by previous surveys [36].

Considering that the CPV-2 vaccine appears to provide a comparatively lower and shorter immunity against heterologous CPVs, there is evidences to suggest that complete immunity may not be provided to dogs even if CPV-2 vaccines are used [37-39]. In many countries, such as Europe, CPV-2a has been overtaken by CPV-2b or CPV-2c, some researches have been to evaluate antigenic relationships among the original canine parvovirus type 2 (CPV-2) and the variants CPV-2a, -2b, and -2c, cross-antigenic evaluation revealed clear differences among the CPV variants [40]. Nevertheless, our study showed that the giant panda and dogs are all new CPV-2a type, while the vaccines available for giant pandas and dogs are CPV-2 type vaccine in China (Nobivac®, Holland) [41]. The effectiveness of CPV-2 vaccine against CPV-2a type has not been evaluated in China.

In sum, wildlife in captive facilities in China is generally not reliably or safely vaccinated. Strain difference between field virus and vaccine candidate virus could be one of the important attributable reasons for some immunization failure. Infectious diseases pose a significant risk to these animals, of which many are endangered species, the mechanisms of the virus into the giant panda population and the adaptation (mutation) of the virus to that species are important topics for future research.

## Materials and methods

### Ethics statement

The animal from which specimens were collected, was handled in accordance with animal protection law of the People’s Republic of China (a draft of an animal protection law in China released on September 18, 2009). This study was approved by the National Institute of Animal Health Animal Care and Use Committee at Sichuan Agricultural University (approval number 2010–020).

### Clinical samples

A total of 36 faecal samples of giant pandas and 97 rectal swabs were collected from dogs suspected to be infected with CPV were gathered simultaneously. Detailed information on the origin and the accession numbers of the CPV-positive samples is shown in Table 2. All activities followed the legal requirements and institutional guidelines set out by the government of P.R. China. The samples were collected in China during a period of 7 months from November 2011 to May 2012. The collected samples were emulsified in 2 ml of 0.1 M PBS of pH 7.4 and centrifuged at 6000 g for 15 min at 4°C. The supernatant was collected and used for PCR amplification.

**Table 2:** Alignment of the deduced amino acid sequences of partial VP2 gene

**Aa residue**	**267**	**297**	**300**	**305**	**324**	**370**	**375**	**426**	**442**
Nt position	3585-3588	3675-3577	3684-3686	3699-3701	3756-3769	3894-3897	3909-3912	4064-4067	4112-4115
M38245(CPV-2)	F	S	A	D	Y	Q	N	N	T
M24003(CPV-2a)	F	S	G	Y	Y	Q	D	N	T
M74849(CPV-2b)	F	S	G	Y	Y	Q	D	D	T
FJ222821(CPV-2c)	F	A	G	Y	Y	Q	D	E	T
AB054215(FPV)	F	A	G	Y	Y	Q	D	N	T
AY742953(New-CPV-2a)	F	A	G	Y	Y	Q	D	N	T
AY742955(New-CPV-2b)	F	A	G	Y	Y	Q	D	D	T
JX624761(A10)	Y	A	G	Y	I	Q	D	N	A
JX624762(A11)	Y	A	G	Y	I	Q	D	N	A
JX624763(A12)	Y	A	G	Y	I	Q	D	N	A
JX624764(B01)	Y	A	G	Y	I	Q	D	N	A
JX624765 (B02)	Y	A	G	Y	I	Q	D	N	A
JX624766(B03)	F	A	G	Y	I	Q	D	N	T
JX624767(B04)	Y	A	G	Y	I	Q	D	N	T
JX624768(B05)	Y	A	G	Y	I	Q	D	N	A
JX624769(B06)	F	A	G	Y	I	Q	D	N	T
JX624770(B07)	Y	A	G	Y	I	Q	D	N	A
JX624771(B11)	F	A	G	Y	I	R	D	N	T
JX624772(B12)	Y	A	G	Y	I	Q	D	N	A

Sequence of one of the representative canine parvovirus field isolate GU139553 (New CPV-2a) is shown aligned with the reference strains M38246 (FPV), M38245 (CPV-2), M24003 (CPV-2a), M74849 (CPV-2b), AY742953 (New CPV-2a), AY742955 (New CPV-2b) and FJ222821 (CPV-2c) obtained from the Genbank.

### Template DNA preparation

Hundred microlitres of the processed supernatant was used for template DNA preparation by boiling at 96°C for 10 min and chilling immediately in crushed ice [42,43]. The supernatants were diluted 1:10 in distilled water to reduce residual inhibitors of DNA polymerase activity [12].

### Primer pair and PCR amplification

PCR amplification was performed using KOD-Plus-Ver.2 (TOYOBO, Japan) and primer pair H_for_ (5′-CAGGTGATGAATTTGCTACA-3′)/H_rev_ (5′-CATTTGGATA -AACTGGTGGT-3′) that amplifies 611 bp fragment of the gene encoding capsid protein [11]. PCR amplification was consisted of 30 cycles of denaturation (95°C 45 s), annealing(51°C 45 s), extension (72°C 45 s) and final extension (72°C 10 min) and the products were analyzed by electrophoresis using 1.5% agarose gel in Tris acetate EDTA (TAE) buffer (1×).

### Sequencing and phylogenetic analysis

PCR products of the correct size (611 bp in length) were amplified and cloned using TArget Clone-Plus-(TOYOBO, Japan),then custom sequenced with primer pair Hfor/Hrev. The sequences were aligned with sequences of prototype CPV strains (M38246-FPV; M38245-CPV-2; M24003-CPV-2a; M74849-CPV-2b; AY742953- New CPV-2a; AY742955-New CPV-2b; FJ222821-CPV-2c) using Clustal W (http://www.clustal.org). The sequences were analyzed with respect to the prototype CPV-2 strain for the nucleotide variation of VP2 gene at positions 3675, 3684, 3699, 3756, 3909 and 4062 with the corresponding amino acid residues at 297, 300, 305, 324, 375 and 426, respectively.

For the phylogenetic analysis, 35 canine parvovirus sequences from various parts of the world were retrieved from the GenBank and used. The sequences were aligned using Clustal W. Maximum likelihood tree was drawn using the MEGA 5.0 Software [44].

## Competing interests

The authors declare that they have no competing interests.

## Authors’ contributions

Conceived and designed the experiments: LG QGY. Performed the experiments: LG SLY ZXH MLZ ZFQ. Analyzed the data: LG SLY MLZ ZXH. Contributed reagents/materials/analysis tools :SJC ZHZ RH YPR CDW XTW SJC WZG. Wrote the paper: LG SLY. All of the authors read and approved the final version of the manuscript.

## References

[B1] Decaro N Buonavoglia C Canine parvovirus—A review of epidemiological and diagnostic aspects, with emphasis on type 2c Vet Microbiol 2012 155 1 12 10.1016/j.vetmic.2011.09.007 21962408 PMC7173204

[B2] Kang BK Song DS Lee CS Jung KI Park SJ Kim EM Park BK Prevalence and genetic characterization of canine parvoviruses in Korea Virus Genes 2008 36 127 133 10.1007/s11262-007-0189-6 18181016

[B3] Decaro N Elia G Campolo M Desario C Lucente M Bellacicco A Buonavoglia C New approaches for the molecular characterization of canine parvovirus type 2 strains J Vet Med B 2005 52 316 319 10.1111/j.1439-0450.2005.00869.x 16316391

[B4] Parrish CR Aquadro CF Strassheim M Evermann J Sgro J Mohammed H Rapid antigenic-type replacement and DNA sequence evolution of canine parvovirus J Virol 1991 65 6544 6552 1942246 10.1128/jvi.65.12.6544-6552.1991 PMC250707

[B5] Parrish CR O’Connell PH Evermann JF Carmichael LE Natural variation of canine parvovirus Science 1985 230 1046 1048 10.1126/science.4059921 4059921

[B6] Parker JSL Murphy WJ Wang D O’Brien SJ Parrish CR Canine and feline parvoviruses can use human or feline transferrin receptors to bind, enter, and infect cells J Virol 2001 75 3896 3902 10.1128/JVI.75.8.3896-3902.2001 11264378 PMC114880

[B7] Truyen U Evolution of canine parvovirus: loss and gain of the feline host] Tierarztl Prax 1996 24 316 8767195

[B8] Martella V Cavalli A Decaro N Elia G Desario C Campolo M Bozzo G Tarsitano E Buonavoglia C Immunogenicity of an intranasally administered modified live canine parvovirus type 2b vaccine in pups with maternally derived antibodies Clin Diagn Lab Immun 2005 12 1243 1245 10.1128/CDLI.12.10.1243-1245.2005 PMC1247831 16210491

[B9] Ohshima T Hisaka M Kawakami K Kishi M Tohya Y Mochizuki M Chronological analysis of canine parvovirus type 2 isolates in Japan J Vet Med Sci Japan Soc Vet Sci 2008 70 769 10.1292/jvms.70.769 18772550

[B10] Truyen U Evolution of canine parvovirus–a need for new vaccines? Vet Microbiol 2006 117 9 13 10.1016/j.vetmic.2006.04.003 16765539

[B11] Buonavoglia C Martella V Pratelli A Tempesta M Cavalli A Buonavoglia D Bozzo G Elia G Decaro N Carmichael L Evidence for evolution of canine parvovirus type 2 in Italy J Gen Virol 2001 82 3021 11714979 10.1099/0022-1317-82-12-3021

[B12] Decaro N Martella V Desario C Bellacicco A Camero M Manna L d’Aloja D Buonavoglia C First detection of canine parvovirus type 2c in pups with haemorrhagic enteritis in Spain J Vet Med B 2006 53 468 472 10.1111/j.1439-0450.2006.00974.x PMC7165763 17123424

[B13] Nakamura M Tohya Y Miyazawa T Mochizuki M Phung H Nguyen N Huynh L Nguyen L Nguyen P Nguyen P A novel antigenic variant of canine parvovirus from a Vietnamese dog Arch Virol 2004 149 2261 2269 10.1007/s00705-004-0367-y 15503211

[B14] Touihri L Bouzid I Daoud R Desario C El Goulli AF Decaro N Ghorbel A Buonavoglia C Bahloul C Molecular characterization of canine parvovirus-2 variants circulating in Tunisia Virus Genes 2009 38 249 258 10.1007/s11262-008-0314-1 19112611

[B15] Decaro N Desario C Addie DD Martella V Vieira MJ Elia G Zicola A Davis C Thompson G Thiry E Molecular epidemiology of canine parvovirus, Europe Emerg Infect Dis 2007 13 1222 1224 10.3201/eid1308.070505 17953097 PMC2828098

[B16] Decaro N Desario C Amorisco F Losurdo M Elia G Parisi A Ventrella G Martella V Buonavoglia C Detection of a canine parvovirus type 2c with a non-coding mutation and its implications for molecular characterisation Vet J 2013 (In Press) 10.1016/j.tvjl.2012.12.017 23375346

[B17] Hong C Decaro N Desario C Tanner P Pardo MC Sanchez S Buonavoglia C Saliki JT Occurrence of canine parvovirus type 2c in the United States J Vet Diagen Invest 2007 19 535 539 10.1177/104063870701900512 17823398

[B18] Wu J Cao GW Jiang YK Luo JM Yang SQ Wu GQ Huang XW He GX Li YS Ye ZY Serosurvey of infection of parvovirus in giant pandas Chin J Preventive Vet Med 1988 2 38 39

[B19] Mainka SA Qiu X He T Appel MJ Serologic survey of giant pandas (Ailuropoda melanoleuca), and domestic dogs and cats in the Wolong Reserve, China J Wildlife Dis 1994 30 86 89 10.7589/0090-3558-30.1.86 8151830

[B20] Loeffler IK Howard J Montali RJ Hayek L-A Dubovi E Zhang Z Yan Q Guo W Wildt DE Serosurvey of ex situ giant pandas (Ailuropoda melanoleuca) and red pandas (Ailurus fulgens) in China with implications for species conservation J Zoo Wildlife Med 2007 38 559 566 10.1638/2006-0008R.1 18229861

[B21] Qin Q Li D Zhang H Hou R Zhang Z Zhang C Zhang J Wei F Serosurvey of selected viruses in captive giant pandas (Ailuropoda melanoleuca) in China Vet Microbiol 2010 142 199 204 10.1016/j.vetmic.2009.09.062 19913371 PMC7117238

[B22] Chinchkar S Mohana Subramanian B Hanumantha Rao N Rangarajan P Thiagarajan D Srinivasan V Analysis of VP2 gene sequences of canine parvovirus isolates in India Arch Virol 2006 151 1881 1887 10.1007/s00705-006-0753-8 16583153

[B23] Clegg S Coyne K Parker J Dawson S Godsall S Pinchbeck G Cripps P Gaskell R Radford A Molecular Epidemiology and Phylogeny Reveal Complex Spatial Dynamics in Areas Where Canine Parvovirus Is Endemic J Virol 2011 85 7892 7899 10.1128/JVI.01576-10 21593180 PMC3147911

[B24] Martella V Decaro N Elia G Buonavoglia C Surveillance activity for canine parvovirus in Italy J Vet Med B 2005 52 312 315 10.1111/j.1439-0450.2005.00875.x 16316390

[B25] Mochizuki M Ohshima T Une Y Yachi A Recombination between vaccine and field strains of Canine Parvovirus is revealed by isolation of virus in canine and feline cell cultures J Vet Med Sci 2008 70 1305 1314 10.1292/jvms.70.1305 19122396

[B26] Decaro N Desario C Billi M Mari V Elia G Cavalli A Martella V Buonavoglia C Western European epidemiological survey for parvovirus and coronavirus infections in dogs Vet J 2011 187 195 199 10.1016/j.tvjl.2009.10.027 19932978 PMC7110566

[B27] Zhang R Yang S Zhang W Zhang T Xie Z Feng H Wang S Xia X Phylogenetic analysis of the VP2 gene of canine parvoviruses circulating in China Virus Genes 2010 40 397 402 10.1007/s11262-010-0466-7 20217205

[B28] Ikeda Y Mochizuki M Naito R Nakamura K Miyazawa T Mikami T Takahashi E Predominance of canine parvovirus (CPV) in unvaccinated cat populations and emergence of new antigenic types of CPVs in cats Virology 2000 278 13 19 10.1006/viro.2000.0653 11112475

[B29] Battilani M Scagliarini A Tisato E Turilli C Jacoboni I Casadio R Prosperi S Analysis of canine parvovirus sequences from wolves and dogs isolated in Italy J Gen Virol 2001 82 1555 1560 11413365 10.1099/0022-1317-82-7-1555

[B30] Jeoung S-Y Ahn S-J Kim D Genetic analysis of VP2 gene of canine parvovirus isolates in Korea J Vet Med Sci Japan Soc Vet Sci 2008 70 719 10.1292/jvms.70.719 18685246

[B31] Decaro N Desario C Parisi A Martella V Lorusso A Miccolupo A Mari V Loredana Colaianni M Cavalli A Di Trani L Genetic analysis of canine parvovirus type 2c Virology 2009 385 5 10 10.1016/j.virol.2008.12.016 19144369

[B32] Hoelzer K Shackelton LA Parrish CR Holmes EC Phylogenetic analysis reveals the emergence, evolution and dispersal of carnivore parvoviruses J Gen Virol 2008 89 2280 2289 10.1099/vir.0.2008/002055-0 18753238 PMC2735869

[B33] Stucker KM Pagan I Cifuente JO Kaelber JT Lillie TD Hafenstein S Holmes EC Parrish CR The role of evolutionary intermediates in the host adaptation of canine parvovirus J Virol 2012 86 1514 1521 10.1128/JVI.06222-11 22114336 PMC3264339

[B34] Tsao J Chapman MS Agbandje M Keller W Smith K Wu H Luo M Smith TJ Rossmann MG Compans RW The three-dimensional structure of canine parvovirus and its functional implications Science 1991 251 1456 1464 10.1126/science.2006420 2006420

[B35] Kaelber JT Demogines A Harbison CE Allison AB Goodman LB Ortega AN Sawyer SL Parrish CR Evolutionary reconstructions of the transferrin receptor of Caniforms supports canine parvovirus being a re-emerged and not a novel pathogen in dogs PLoS Pathog 2012 8 e1002666 10.1371/journal.ppat.1002666 22570610 PMC3342950

[B36] Pereira CAD Leal ES Durigon EL Selective regimen shift and demographic growth increase associated with the emergence of high-fitness variants of canine parvovirus Infect Genet Evol 2007 7 399 409 10.1016/j.meegid.2006.03.007 16716762

[B37] Elia G Cavalli A Cirone F Lorusso E Camero M Buonavoglia D Tempesta M Antibody levels and protection to canine parvovirus type 2 J Vet Med B 2005 52 320 322 10.1111/j.1439-0450.2005.00870.x 16316392

[B38] Decaro N Cirone F Desario C Elia G Lorusso E Colaianni M Martella V Buonavoglia C Severe parvovirus in a 12-year-old dog that had been repeatedly vaccinated Vet Rec 2009 164 593 595 10.1136/vr.164.19.593 19429938

[B39] Decaro N Desario C Elia G Martella V Mari V Lavazza A Nardi M Buonavoglia C Evidence for immunisation failure in vaccinated adult dogs infected with canine parvovirus type 2c Microbiol-Quart J Microbiol Sci 2008 31 125 130 18437851

[B40] Cavalli A Martella V Desario C Camero M Bellacicco AL De Palo P Decaro N Elia G Buonavoglia C Evaluation of the antigenic relationships among canine parvovirus type 2 variants Clin Vaccine Immunol 2008 15 534 539 10.1128/CVI.00444-07 18160619 PMC2268271

[B41] Wang CD Zhang ZH Current status of canine distemper vaccine immune among giant pandas and red pandas Sichuan J Zool 2006 25 668 672

[B42] Decaro N Desario C Campolo M Elia G Martella V Ricci D Lorusso E Buonavoglia C Clinical and virological findings in pups naturally infected by canine parvovirus type 2 Glu-426 mutant J Vet Diagen Invest 2005 17 133 10.1177/104063870501700206 15825493

[B43] Schunck B Kraft W Truyen U A simple touch-down polymerase chain reaction for the detection of canine parvovirus and feline panleukopenia virus in feces J Virol Met 1995 55 427 433 10.1016/0166-0934(95)00069-3 8609207

[B44] Tamura K Peterson D Peterson N Stecher G Nei M Kumar S MEGA5: molecular evolutionary genetics analysis using maximum likelihood, evolutionary distance, and maximum parsimony methods Mol Biol Evol 2011 28 2731 2739 10.1093/molbev/msr121 21546353 PMC3203626

